# Ab initio study for molecular-scale adsorption, decomposition and desorption on AlN surfaces during MOCVD growth

**DOI:** 10.1038/s41598-020-72973-w

**Published:** 2020-10-20

**Authors:** Jiadai An, Xianying Dai, Runqiu Guo, Lansheng Feng, Tianlong Zhao

**Affiliations:** 1 School of Microelectronics, Xidian University, Xi’an, 710071 China; 2 State Key Discipline Laboratory of Wide Bandgap Semiconductor Technologies, Xidian University, Xi’an, 710071 China; 3 School of Mechano-Electronic Engineering, Xidian University, Xi’an, 710071 China

**Keywords:** Materials science, Theory and computation

## Abstract

Since AlGaN offers new opportunities for the development of the solid state ultraviolet (UV) luminescence, detectors and high-power electronic devices, the growth of AlN buffer substrate is concerned. However, the growth of AlN buffer substrate during MOCVD is regulated by an intricate interplay of gas-phase and surface reactions that are beyond the resolution of experimental techniques, especially the surface growth process. We used density-functional ab initio calculations to analyze the adsorption, decomposition and desorption of group-III and group-V sources on AlN surfaces during MOCVD growth in molecular-scale. For AlCH_3_ molecule the group-III source, the results indicate that AlCH_3_ is more easily adsorbed on AlN (0001) than (000$\overline{1}$) surface on the top site. For the group-V source decomposition we found that NH_2_ molecule is the most favorable adsorption source and adsorbed on the top site. We investigated the adsorption of group-III source on the reconstructed AlN (0001) surface which demonstrates that NH_2_-rich condition has a repulsion effect to it. Furthermore, the desorption path of group-III and group-V radicals has been proposed. Our study explained the molecular-scale surface reaction mechanism of AlN during MOCVD and established the surface growth model on AlN (0001) surface.

## Introduction

Since high-Al-content AlGaN can achieve a wide range of adjustable direct bandgap, high temperature and high pressure resistance and other properties, AlGaN has attracted much attention as a key material in the fields of solid state ultraviolet (UV) luminescence, detectors and high-power electronic devices^1–4^. Studies have shown that there is a large lattice mismatch and thermal mismatch between the AlGaN and sapphire substrate. Due to the mismatch there will generate some non-radiative composite center which affects the luminous efficiency of the material^5–7^. In order to reduce the problem caused by substrate mismatch, the AlN buffer substrate usually be used to grow high-Al-content A1GaN epitaxial film materials. Therefore, we need to study the growth mechanism of AlN to grow high-quality AlN layers firstly^8–10^.

The growth of high-quality AlN layers has been intensively performed by means of epitaxial growth, and the optimization of epitaxial growth condition is an important factor to improve crystal quality^11–13^. Theoretical studies on the epitaxial growth mechanisms of AlN are sparse and focus on the atomic and electronic structure^13–16^, and some surface growth mechanisms have been studied in atom-scale^10,17–20^, but the molecular-scale growth processes on AlN polar surfaces during epitaxial growth still remain unclear^21,22^. In this work, the adsorption, decomposition and desorption processes of group-III and group-V sources on AlN polar surfaces during MOCVD growth in molecular-scale were investigated to establish the initial growth model on the surface.

## Calculation modeling

**Figure 1:**
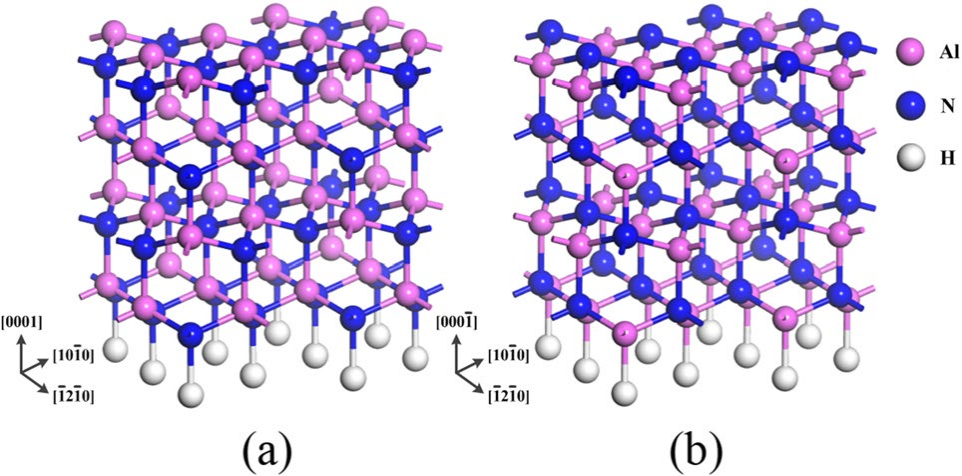
The models of (2 × 2) slab models with four AlN layers (**a**) AlN (0001) surface the top layer is Al atoms and (**b**) AlN (000$\overline{1}$) surface the top layer is N atoms. The bottom layer of these models is terminated with fictitious hydrogen atoms, where pink, blue and white spheres denote aluminum, nitrogen and hydrogen atoms, respectively.

Due to gas-phase chemical reaction, the surface reaction precursors reaching high-temperature substrate are mainly AlCH_3_ molecules generated from decomposition of metallic organic compounds such as Al(CH_3_)_3_, which are used as the group-III sources to investigate the reaction process on AlN surface^23^. The models used for AlN (0001) and AlN (000$\overline{1}$) surfaces were (2 × 2) slab models with four AlN bilayers and are shown in Fig. 1. The vacuum region above the surface was set to 20 Å^24,25^. In order to maintain the crystal structure of the bulk AlN, the coordinates of the bottom four layers and the fictional H atom were fixed, which used H atoms with 0.75 atomic number and 1.25 atomic number for AlN (0001) and AlN (000$\overline{1}$) surfaces, respectively. While those of the top four layers were variable^26^.

**Figure 2:**
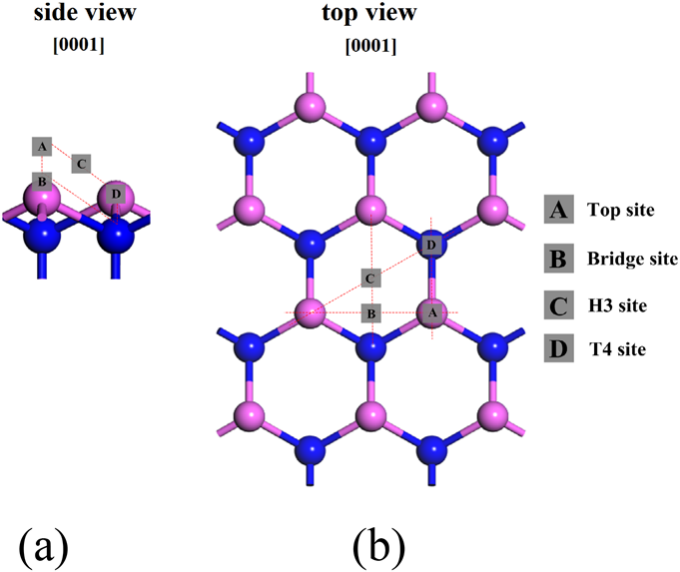
(**a**) Side-view and (**b**) Top-view of (2 × 2) slab model of the AlN (0001) surface. The signs show the adsorption sites for III-group and V-group sources, where pink and blue spheres denote aluminum and nitrogen atoms, respectively.

The group-III and group-V sources were gradually moved from the vacuum space of the model to the adsorption sites shown in Fig. 2, and the adsorption energy was calculated for each case. In order to compare the stability of surface after adsorption, the surface formation energy was calculated using the chemical potential method^27–29^. The van der Waals (vdW) dispersion interactions are a key ingredient for molecule adsorption. For the calculation, the density functional theory (DFT)-D2/D3 with a generalized gradient approximation (GGA) for the exchange correlation energy^26,30,31^. The function used was a revised Perdew-Burke-Ernzerh of function (RPBE)^26,32–35^. The wave functions were expanded in terms of numerical basis sets. The real-space cutoff energy was set as 600 eV, and 6 × 4 × 2 k-point sampling was used. The accuracy of this setting is sufficient to meet the requirements of AlN geometric optimization and energy optimization. The calculations were carried out using the program package CASTEP^36,37^. The adsorption energy of molecules adsorbed on the surface of AlN can be obtained by the total energy difference:

1 $$ E_{ad} = E_{total} - E_{molecules} - E_{slab} $$

where $E_{{{\text{total}}}}$ refers to the total energy of the optimized AlN layers with the adsorbed molecules. $E_{molecules}$ and $E_{{{\text{slab}}}}$ represent the energy of the molecules computed in the gas phase and the optimized slab of the AlN layers without adsorption, respectively^32^.

To study the desorption process of adsorbed molecules, we searched the transition states and found the most stable and the minimum energy by calculating reaction energy barrier used a LST/QST calculation method based on the transition state theory of DFT. During the deposition and growth on the surface of high temperature MOCVD, the reaction path direction was along the vertical direction to the growth surface (normal to the surface)^38,39^.

## Results and discussion

### Adsorption of group-III source on AlN surface

**Figure 3:**
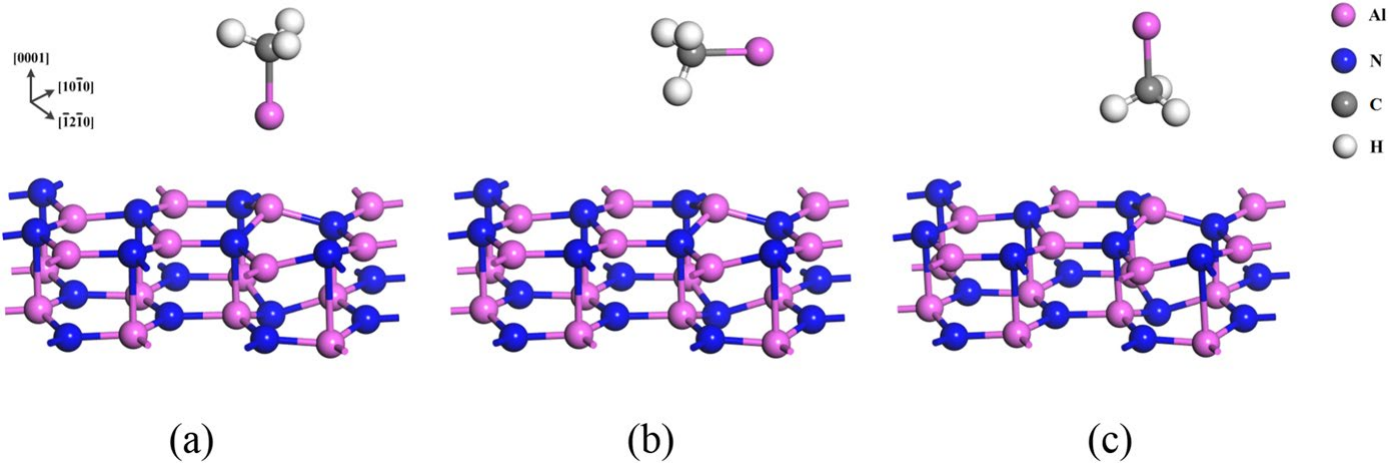
The configurations for AlCH_3_ molecule adsorption to the AlN (0001) surface; (**a**) Al atom faces to the surface, (**b**) AlCH_3_ molecule lies parallel to the surface and (**c**) H atom faces to the surface, where pink, blue and white spheres denote aluminum, nitrogen and hydrogen atoms, respectively.

Firstly, the structures that group-III source adsorbed on the adsorption site on AlN (0001) and AlN (000$\overline{1}$) surfaces (shown in Fig. 2) were optimized. In the case of AlCH_3_ molecule, three configurations can be considered, which are shown in Fig. 3. Geometry optimization was performed for these configurations and it was found that AlCH_3_ molecule adsorbed with the configuration which Al atom faces to surface.

Based on the surface structures, the calculation for the adsorption process was performed for AlCH_3_, and the results are shown in Table 1. On AlN (0001) surface, it was found that the minimum distance of Al atom in AlCH_3_ molecule with the topmost surface occurs in the case of top site with the value of 2.667 Å. For the angle between the C–Al bond and the topmost surface, the top site with the value of 1.097 degree shows the smallest angle. And the adsorption energy of the top site was the lowest with the value of − 4.58 eV. The results indicated that AlCH_3_ will diffusion to the top site when it reaches on AlN (0001) surface. On the contrary, on the AlN (000$\overline{1}$) surface, the adsorption energies of all the sites are positive, indicating that the adsorption process requires heat which is not stable.

**Table 1:** Structure parameters, stable adsorption sites at AlCH_3_ adsorbed on the AlN (0001) and AlN (000$\overline{1}$) surfaces, and the corresponding adsorption energies.

Layer	Initial site	Final site	$E_{ad}$(eV)	$d_{Al - surf}$(Å)	$\theta$(degree)
AlCH_3_ on the AlN (0001) surface	Top	Top	− 4.58	2.667	1.097
	Bridge	Top	− 0.21	2.732	51.049
	h3	Top	− 0.319	2.720	73.192
	t4	Top	0.135	3.002	49.201
AlCH_3_ on the AlN (000$\overline{1}$) surface	Top	t4	4.764	1.182	48.442
	Bridge	h3	3.562	1.172	20.754
	h3	h3	4.125	0.957	1.098
	t4	h3	3.341	1.165	74.075

$E_{ad}$ represents the adsorption energy. $d_{Al - surf}$ represents the average distance of Al atom in AlCH_3_ with the topmost surface. $\theta$ represents the angle between the C–Al bond and the topmost surface.

**Figure 4:**
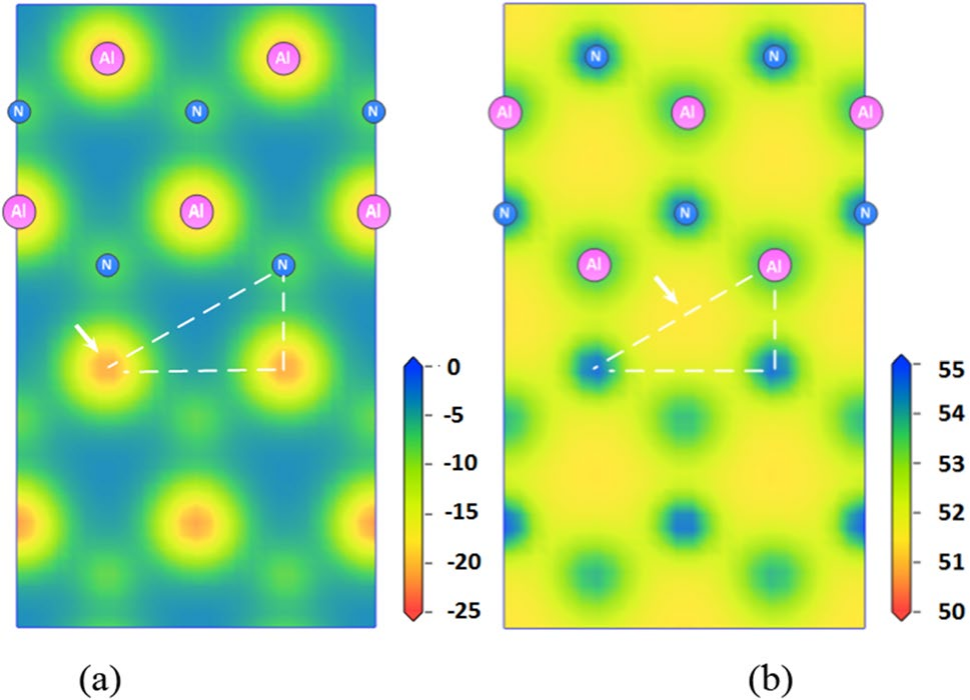
Contour plots of the PES for the AlCH_3_ molecule on the (**a**) AlN (0001) surface and (**b**) AlN (000$\overline{1}$) surface, the unit is eV.

The calculation of the potential-energy surfaces (PES) for AlCH_3_ molecules on the 2 × 2 AlN (0001) and AlN (000$\overline{1}$) surfaces are shown as Fig. 4. The most stable adsorption site on AlN (0001) surface is located above the topmost surface Al atom which is the top site ((arrow in Fig. 4a). This results in the formation of an Al–Al bond (bond length 2.66 Å) between Al atom in AlCH_3_ molecule and topmost surface Al atom. The adsorption energy *E*_*ad*_ = − 4.58 eV, corresponding to the energy gain to form an Al-Al bond. Similarly, the most stable adsorption site on the AlN (000$\overline{1}$) surface is located above the center of the hexagonal structure on topmost surface which is the h3 site ((arrow in Fig. 4b). As shown in Fig. 4b, consistent with the adsorption energy calculated above, the adsorption energy of AlN (000$\overline{1}$) surface is positive which indicates the adsorption process is not stable.

The adsorption of AlCH_3_ molecule on AlN (0001) surface is bound to be accompanied by the transfer of charge between atoms and the change of electronic structure. Therefore, the Mulliken charge population of the adsorbed particles and the topmost surface atoms is analyzed, and the results are shown in Table 2. Due to the three H atoms in CH_3_ are symmetrically distributed, the Mulliken charge population of one is listed only. As the electrons in the inner layer of an atom are stable, the s and p orbitals in Table 2 show the outermost atomic orbitals. In Table 2, there is a slight change of the charge population numbers of C and H atoms before and after adsorption, while the charge population numbers of s and p orbitals of Al atom in AlCH_3_ molecule and Al atom on the top site of topmost surface changed greatly. The charge population of s and p orbitals of Al atom in AlCH_3_ molecule changed from 1.81 and 0.59 before adsorption to 1.52 and 0.70 after adsorption, the electron transfer number is 0.29 and 0.11, respectively. Similarly, the electron transfer number of s and p orbitals of Al atom on the top site is 0.12 and -0.05, respectively. Therefore, the adsorption of AlCH_3_ molecule on the AlN (0001) surface mainly depends on the interaction between the s and p orbitals of Al atom in AlCH_3_ molecule and the Al atom on the top site of topmost surface. The change of charge shows that the increase of positivity of Al atom in AlCH_3_ molecule after adsorption is greater than that in the Al atom on the top site, indicating that Al atom in AlCH_3_ loses electrons in the adsorption process, and the adsorption mechanism is that the adatoms transfer electrons to the surface atoms.

**Table 2:** Mulliken charge population of each atoms in AlCH_3_ and Al atom on the top site of topmost surface before and after adsorption.

	Atoms	s	p	Charge (e)
Before adsorption	C	1.55	3.76	− 1.32
	H	0.76	0.00	0.24
	Al	1.81	0.59	0.60
	AlCH_3_	none	none	0.00
	Al_surf_	0.74	1.08	1.18
After adsorption (top site)	C	1.54	3.75	− 1.29
	H	0.76	0.00	0.24
	Al	1.52	0.70	0.78
	AlCH_3_	none	none	0.22
	Al_surf_	0.62	1.13	1.26

The results indicated that the adsorption energy and the stable adsorption site of group-III source AlCH_3_ molecule on AlN surface are affected by the topmost surface atoms, and the AlCH_3_ molecule is easier adsorbed on the top site of AlN (0001) surface which explained in theory why the epitaxial growth of AlN for devices has usually been grown along the [0001] direction.

### Decomposition and adsorption of group-V sources on AlN surface

According to the above analysis, the AlN has been mainly grown along the [0001] direction, so of this part the growth surface is on AlN (0001) surface. First, the structures that NH_n_ (n = 0–3) adsorbed on the adsorption sites on AlN (0001) surface (shown in Fig. 2) were optimized. The adsorption sites and adsorption energies for each adsorption species are shown in Table 3. From the calculations, it was shown that NH_3_, NH_2_, NH and N favorably were adsorbed on the top, top, bridge and t4 sites on AlN (0001) surface, respectively. It was found that the adsorption energy of NH_3_ is positive, and $d_{N - surf}$ and $\theta$ were the maximum with the value of 2.461 Å and 0.069 degree which indicates the NH_3_ is not stable species. From the adsorption energy, N is the most favorable adsorption species. However, these adsorption energies were obtained on the assumption that NH_2_, NH and N were present in the vapor phase from the beginning. In addition, we should consider the decomposition of nitrogen sources under high temperature gas phase. Therefore, we calculated the energy required for the decomposition process of the repeated dehydrogenation from the NH_3_.

2 $$ NH_{3} = NH_{2} + H $$

3 $$ NH_{2} = NH + H $$

4 $$ NH = N + H $$

**Table 3:** Structure parameters, stable adsorption sites at NH_n_ adsorbed on the AlN (0001) surface and the corresponding adsorption energies.

Adsorption species	Adsorption site	$E_{ad}$ (eV)	$d_{N - surf}$ (Å)	$\theta$ (degree)
NH_3_	Top	1.926	2.461	0.069
	Bridge	2.876	3.121	0.122
	H3	2.012	2.975	0.091
	T4	2.351	3.001	0.103
NH_2_	Top	− 4.325	1.981	0.022
	Bridge	− 3.781	2.031	0.211
	H3	− 3.564	2.001	0.199
	T4	− 2.961	2.155	0.200
NH	Top	− 5.784	1.699	0.054
	Bridge	− 6.436	0.556	0.034
	H3	− 5.011	1.800	0.061
	T4	− 5.013	1.801	0.059
N	Top	− 7.011	1.021	none
	Bridge	− 6.787	1.313	none
	H3	− 6.996	0.997	none
	T4	− 7.353	0.972	none

$E_{ad}$ represents the adsorption energy. $d_{N - surf}$ represents the average distance of N atoms of NH_n_ with the surface atoms. $\theta$ represents the angle between the NH_n_ and the AlN (0001) surface.

The obtained decomposition energy for reactions (2), (3) and (4) were 2.01, 3.59 and 1.61 eV, respectively. Therefore, the values of the reaction energies of NH_3_, NH_2_, NH and N adsorption on the surface are 1.926, − 2.315, − 0.836, − 0.143 eV, respectively. This result suggests that NH_2_ is the most favorable adsorption species and on the top site is the most stable when the decomposition of NH_3_ is considered.

### Adsorption of group-III source on reconstructed AlN (0001) surface

**Figure 5:**
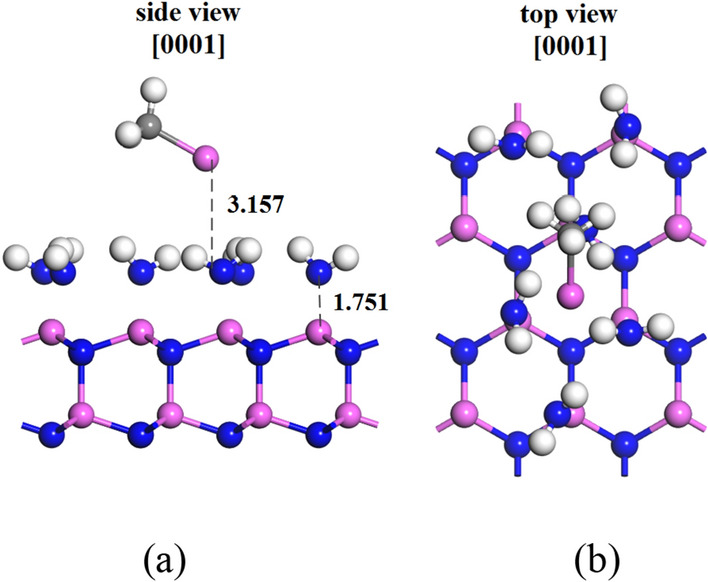
(**a**) Side-view and (**b**) Top-view of (2 × 2) slab model of reconstructed AlN (0001) surface, where pink, blue and white spheres denote aluminum, nitrogen and hydrogen atoms, respectively. The digital signs show the distance from AlCH_3_ to the NH_2_ coverage layers and the distance from the NH_2_ coverage layers to the AlN (0001) surface, the unit is Å.

Since surface reconstructions affect the crystals morphology and play an important role to fabricate high-quality crystals, understanding surface reconstructions is an important issue^21,22^. The adsorption on AlN (0001) surface under N-rich conditions is much easier than that under H-rich conditions, which the N-rich and H-rich conditions refer to the N atom and H atom coverage on the surface^10^. On the results of decomposition and adsorption of group V source on AlN (0001) surface, we investigate the adsorption on reconstruction AlN (0001) surface with NH_2_ as coverage source. The structure that AlCH_3_ molecule adsorb to reconstructed AlN (0001) surface (shown in Fig. 5) was optimized and the adsorption energy was calculated. As shown in the Fig. 5, we can see that the AlCH_3_ moved from top site to h3 site, and the distance from AlCH_3_ to the reconstructed AlN (0001) surface is 3.157 Å larger than that on the AlN (0001) surface. Meanwhile, the optimized adsorption energy of AlCH_3_ molecule is 5.78 eV, indicating that the adsorption process requires heat which is not stable on reconstructed AlN (0001) surface. Al atom in AlCH_3_ molecule is not bonded with the N and H atoms in NH_2_ the surface covering layer. While The N atom in each NH_2_ molecule in the covering layer forms a Al–N covalent bond with Al atom on the top site of topmost surface, but the covalency is very weak. These results indicate that the NH_2_ coverage layer have a repulsion effect to AlCH_3_ molecule adsorption. There is a research has shown that H atoms tend to desorb from AlN (0001) surface even under high H_2_ pressures^37^, and Toru Akiyama et al. reported, the N atoms coverage layer is easier to promote the growth of AlN on AlN (0001) surface than that of H atoms during the MOVPE^10^. In summary, we have found that when N and H atoms cover the surface in the form of NH_2_ molecular structure, they will inhibit the adsorption of Al source on AlN (0001) surface, which also indicated that the desorption of H atom has a greater impact on the initial surface growth process on AlN (0001) surface than the adsorption of N atom.

### Desorption path of group-III and group-V sources on AlN surface

**Figure 6:**
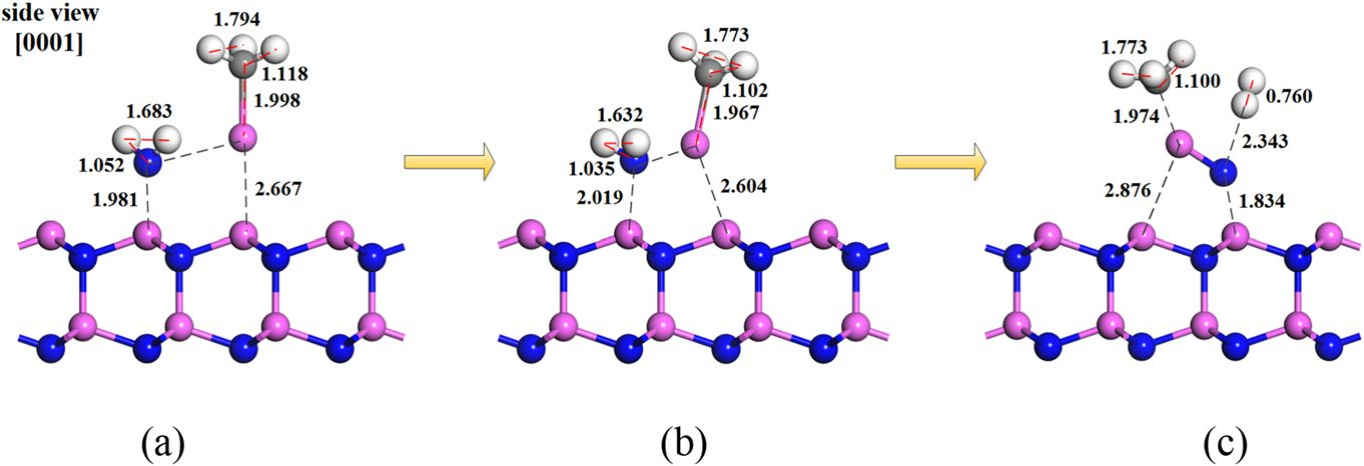
The desorption process of AlCH_3_ and NH_2_ molecules on the AlN (0001) surface; (**a**) Initial adsorption, (**b**) Stable adsorption and (**c**) Desorption state, where pink, blue and white spheres denote aluminum, nitrogen and hydrogen atoms, respectively. The digital signs show the distances, the unit is Å.

Our study shows that the adsorption of group-III and group-V sources on the AlN (0001) surface is in the form of AlCH_3_ and NH_2_, and the stable adsorption site is top site. In order to established the surface growth model of AlN film, we also modeled the desorption process of group-III and group-V sources on the AlN (0001) surface. The model for the desorption path of group-III and group-V sources on AlN (0001) surface were performed as AlCH_3_ and NH_2_, and the results are shown in Fig. 6. The reaction path (5) shows the optimized desorption path after the AlCH_3_ and NH_2_ molecules adsorbed on AlN (0001) surface top site.

5 $$ {\text{MMAl}}\left( {\text{S}} \right) + {\text{NH}}_{{2}} ({\text{S}} \to {\text{AlN}}\left( {\text{B}} \right) + {\text{OPENA}}\left( {\text{S}} \right) + {\text{OPENN}}\left( {\text{S}} \right) + {\text{CH}}_{{3}} + {\text{H}}_{{2}} $$

where the MMAl(S) and NH_2_(S) refer to the adsorbed AlCH_3_ and NH_2_ molecules on AlN (0001) surface top site. The AlN(B) represents the AlN molecule adsorbed on the topmost on AlN (0001) surface after the desorption process. The OPENA(S) and OPENN(S) are open aluminum and nitrogen sites, respectively. The CH_3_ and H_2_ are the desorption products.

**Figure 7:**
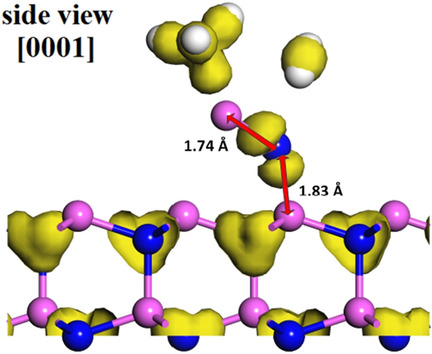
The difference charge density of the initial growth modeling on AlN(0001) surface after the desorption path (5). Yellow regions represent larger charge density.

The energy of reaction path (5) is 2.261 eV. First, the AlCH_3_ and NH_2_ molecules adsorbed on AlN (0001) surface top site, as shown in Fig. 6a. Second, due to the molecular interaction, the molecules stable adsorption sites will be optimized, as shown in Fig. 6b. Finally, the desorption path is the demethylation of AlCH_3_ and dehydrogenation of NH_2_, which the CH_3_ and H_2_ in the gas phase, as shown in Fig. 6c. The charge difference diagram is shown in Fig. 7. The Al atom in AlCH_3_ molecule and N atom in NH_2_ form a Al-N covalent bond with value of 1.74 Å (arrow in Fig. 7), which is shorter than the Al-N bond on AlN (0001) surface. In addition, the N atom in NH_2_ and Al atom on the top site of topmost surface forms a Al-N covalent bond with value of 1.83 Å (arrow in Fig. 7). Therefore, we proposed the surface growth model on AlN (0001) surface as follows: after AlCH_3_ and NH_2_ molecules adsorbed on the top site respectively, they will be follow the desorption path (5) and the initial bonding process is given.

## Conclusions

In summary, the adsorption, decomposition and desorption process of group-III and group-V sources on AlN surfaces during MOCVD growth were investigated using density-functional ab initio calculations in molecular-scale. We have found that AlCH_3_ and NH_2_ molecules prefer to be adsorbed on AlN (0001) surface on the top site. By the study of the adsorption on reconstruction AlN (0001) surface, it follows that AlCH_3_ molecules growth to be prominent under ideal surface rather than NH_2_-rich conditions. Moreover, we have proposed the desorption path of the group-III and group-V sources on AlN (0001) surface. To sum up, the study in this paper explained the molecular-scale surface growth mechanism of AlN during MOCVD, and established the surface growth model on AlN (0001) surface. The results are helpful for the future calculation concerning more detailed growth process of AlN process during MOCVD growth.
